# Prognostic and therapeutic implications of measurable residual disease in acute myeloid leukemia

**DOI:** 10.1186/s13045-021-01148-5

**Published:** 2021-09-03

**Authors:** Marisa J. L. Aitken, Farhad Ravandi, Keyur P. Patel, Nicholas J. Short

**Affiliations:** 1 Department of Leukemia, The University of Texas MD Anderson Cancer Center, Houston, TX USA; 2 McGovern Medical School, UT Health Science Center-Houston, Houston, TX USA; 3 Department of Hematopathology, The University of Texas MD Anderson Cancer Center, Houston, TX USA; 4 Present Address: Department of Internal Medicine, University of Michigan, Ann Arbor, MI USA

**Keywords:** Measurable residual disease, Acute myeloid leukemia, Surrogate endpoint

## Abstract

Quantification of measurable residual disease (MRD) provides critical prognostic information in acute myeloid leukemia (AML). A variety of platforms exist for MRD detection, varying in their sensitivity and applicability to individual patients. MRD detected by quantitative polymerase chain reaction, multiparameter flow cytometry, or next-generation sequencing has prognostic implications in various subsets of AML and at various times throughout treatment. While it is overwhelmingly evident that minute levels of remnant disease confer increased risk of relapse and shortened survival, the therapeutic implications of MRD remain less clear. The use of MRD as a guide to selecting the most optimal post-remission therapy, including hematopoietic stem cell transplant or maintenance therapy with hypomethylating agents, small molecule inhibitors, or immunotherapy is an area of active investigation. In addition, whether there are sufficient data to use MRD negativity as a surrogate endpoint in clinical trial development is controversial. In this review, we will critically examine the methods used to detect MRD, its role as a prognostic biomarker, MRD-directed therapeutics, and its potential role as a study endpoint.

## Introduction

Acute myeloid leukemia (AML) encompasses a heterogeneous group of diseases driven by any number or combination of recurrent mutations, chromosomal aberrations, and/or altered signaling pathways [1]. Most patients with AML achieve remission following induction therapy. However, relapse is common, and with each relapse comes a progressively decreased probability of long-term survival [2, 3]. Leukemic relapse invariably arises from a pre-existing—or at least a closely genetically-related—clone, with only rare exception [4, 5]. Thus, eradication of even the tiniest remnants of disease after therapy is likely to be a requirement for cure in AML. Identification of these remaining leukemia cells, termed measurable residual disease (MRD), is strongly prognostic for clinical outcomes and may have therapeutic implications in the management of AML.

Complete remission (CR) in AML is currently defined by the European LeukemiaNet (ELN) guidelines as a morphologic leukemia-free assessment of bone marrow (BM)—i.e., < 5% blasts and no Auer rods—coupled with no extramedullary disease, and complete recovery of neutrophils (> 1 × 10^9^/L) and platelets (> 100 × 10^9^/L) without exogenous growth factor support [6, 7]. While 60–85% of patients under 60 years of age achieve CR after induction therapy, the cure rate is substantially lower, indicated by the 5-year overall survival (OS) of 40–50% even among patients who respond to induction chemotherapy [1]. In patients older than 60 years, outcomes are even more dismal. These data underlie the now widely accepted notion that morphologic BM assessment is an insufficient determinant of relapse risk, and perhaps the term “complete remission,” as historically defined, is a misnomer. An ever-accumulating body of evidence demonstrates that detectable MRD while in CR is associated with higher relapse risk and shorter survival, and provides additional prognostic information to traditional morphologic response assessment [8–14]. Fittingly, the AML 2017 ELN recommendations defined a new response criterion, which is now considered the optimal response in AML therapy: CR without MRD, or CR_MRD-_ [7].

Despite the undeniable utility of MRD as a prognostic indicator, there is little consensus regarding its utility to guide treatment decisions. In this review, we summarize the current literature on MRD in AML, including methodology of MRD assessment in various AML subsets and its prognostic implications. We also discuss conceptual considerations of how to optimally use MRD to guide clinical decision making and clinical trial design.

## Methodologies for MRD assessment

Given the heterogeneous and oligoclonal nature of AML, a variety of techniques to assess MRD is needed. Platforms for MRD assessment differ primarily in their sensitivity and the population of cases for which they are useful (Table 1). We consider these methods in two major categories: (1) useful in select cases and (2) useful in nearly all cases.

**Table 1:** Features of MRD detection methods

Platform	Case applicability	Sensitivity^a^	Advantages (+)/disadvantages (−)
Karyotyping	~ 50%	1/20	+ Widely available + Well-standardized − Slow turnaround time − Labor intensive − Requires pre-existing abnormal karyotype
FISH	~ 50%	1/100	+ Useful for numeric cytogenetic abnormalities + Relatively quick turnaround time − Labor intensive − Requires pre-existing abnormal karyotype
RT-qPCR	~ 40–50%	1/10,000–1/1,000,000	+ Widely available + Well-standardized + Relatively inexpensive − Single gene assessed per assay − Mutations occurring outside of primer-spanning regions of gene will be missed
MFC	Almost all	1/1,000–1/100,000	+ Widely available + Relatively quick turnaround time + Widely applicable − Not fully standardized − Analysis and interpretation require high-level expertize
NGS	> 95%	1/100–1/1,000,000	+ Simultaneous assessment of numerous targets + Can detect mutations in any sequenced portion of a gene + Very widely applicable − Not widely available − Slow turnaround time − Not standardized − Expensive (particularly to achieve high sensitivity) − Analysis and interpretation require high-level expertize

*FISH* fluorescence in situ hybridization, *MFC* multiparameter flow cytometry, *RT-qPCR* reverse transcription-quantitative polymerase chain reaction, *NGS* next-generation sequencing

^a^Sensitivity is defined as the ability of the assay to reliably detect 1 leukemia-associated target among a maximum of X targets (e.g., some MFC assays can detect 1 leukemic cell among up to 100,000 nucleated cells)

### Methods useful in select cases

#### Karyotyping and fluorescence in situ hybridization (FISH)

Despite their important role at diagnosis, the use of karyotyping and/or FISH for MRD detection is limited, owing in large part to their relative insensitivity. Conventional karyotyping has a sensitivity of only ~ 5% while the sensitivity of FISH is ~ 1% [15]. In addition, karyotype analysis is helpful only when an abnormal karyotype is identified at baseline, rendering this method irrelevant in the approximately 50% of patients with cytogenetically normal AML [16, 17]. The insensitivity of karyotyping is exemplified by observations that despite being in CR with no detectable cytogenetic abnormalities, many patients (up to 50% in some studies) still relapse [18]. Nonetheless, persistence or acquisition of an abnormal karyotype has long been associated with higher relapse rate and shortened survival [19–22]. Detection of persistent leukemia-associated karyotypes in CR is thus strongly suggestive that residual leukemia cells are present in a background of apparently normal morphology. Fortunately, more reliable and discriminative approaches for MRD assessment are available in the modern era.

#### Reverse transcription-quantitative polymerase chain reaction (RT-qPCR)

RT-qPCR is a robust platform for detecting and quantifying recurrent genomic alterations. However, its clinical usefulness as a tool for MRD assessment requires that the detected aberration be stable throughout disease while also representing truly residual disease—not merely a preleukemic clone (e.g., a mutation associated with clonal hematopoiesis of indeterminate potential, CHIP) or a differentiated cell retaining the genomic alteration. In the context of MRD assessment in AML, RT-qPCR is therefore used primarily to detect fusion transcripts: *PML-RARA* in acute promyelocytic leukemia (APL), *RUNX1-RUNX1T1* and *CBF-SMMHC* found in core-binding factor (CBF) AML, or recurrent mutations such as those in *NPM1*, all of which represent founding genomic lesions in AML*.* Outside of these subsets, RT-qPCR is not routinely recommended for MRD monitoring [23]. Unfortunately, this limits the applicability of RT-qPCR for MRD evaluation to less than half of adult AML cases [15].

RT-qPCR is a highly sensitive method of detection, with sensitivities ranging from 10^–4^ to 10^–5^ in most cases (and down to nearly 10^–6^ if adequate genetic material is available) [24]. In addition, it is a highly standardized platform. Most results are normalized to expression of another gene (e.g., *ABL1*) with degradation kinetics similar to the transcript of interest. Reporting results in this way accounts for RNA degradation that occurs during sample processing, allowing for sound comparison between samples [25]. Digital droplet PCR (ddPCR) is an emerging technique with higher sensitivity and more robust absolute quantification than RT-qPCR, though the additional clinical value of this technique remains to be seen compared to standard RT-qPCR [26–28].

### Methods useful in nearly all cases

#### Multiparameter flow cytometry (MFC)

MFC uses fluorochrome-conjugated antibodies to identify anomalous patterns of protein expression on leukemic blasts. Analysis of these data includes assessment of “difference from normal” (DfN), leukemia-associated immunophenotypes (LAIPs), or a combination thereof. The DfN approach compares a remission sample immunophenotype to pre-defined, stereotypical antigen expression on normal hematopoietic cells and therefore does not require a diagnostic sample. In contrast, assessment of LAIPs defines a specific aberrant leukemia-associated protein expression pattern at diagnosis and tracks this immunophenotype in follow-up samples. These LAIPs may be characterized by co-expression of mature and immature antigens (e.g., mature cell markers on immature cells), lineage infidelity (e.g., lymphoid markers on myeloid cells), or abnormal amounts of a normal antigen within a cell subset (e.g., overabundance of normal markers) [29]. Combining these two approaches can account for phenotypic shift over time while also maintaining patient-specific considerations.

MFC is generally able to detect 1 abnormal cell in about 10^4^ cells [29]. Compared to other platforms, MFC is also rather quick, usually providing MRD information within hours, rather than on the order of days, as is required for most molecular-based MRD assessments. MFC can also be used to detect expression of leukemia stem cell (LSC) markers, including CLL1, CD123, CD200, and others [30–33], and some studies suggest that detection of LSCs within MRD is an adverse prognostic factor [31, 34, 35]. While antibody panels are increasingly standardized, interpretation of MFC requires an experienced pathologist, and inter-laboratory differences in interpretation are not uncommon [23].

#### Next-generation sequencing (NGS)

NGS can be used to perform simultaneous assessment of genes in a targeted or global manner. In the current era, targeted NGS is critical for disease classification and prognostication [36]. In the context of MRD assessment, targeted NGS is commonly used for serial assessment of mutations found at diagnosis. The interpretation of these mutations as MRD must be performed with caution, as several AML-associated mutations (e.g., *DNMT3A, TET2, ASXL1*) are associated with CHIP, and may not necessarily represent true leukemia in the setting of MRD [13, 37, 38]. At least one driver mutation can be identified in 96% of patients with de novo AML, and 86% of patients have two or more driver mutations [39]. Large, targeted NGS panels are therefore likely to be a useful MRD tool for the vast majority of patients, particularly as the number of genes assessed in targeted panels increases. In contrast to RT-qPCR, NGS also has the added benefit of detecting any mutation in a sequenced portion of a gene—beyond the targeted, most commonly mutated areas flanked by PCR primers [13, 40, 41].

While NGS theoretically can achieve a much higher sensitivity than RT-qPCR or MFC, the most commonly used clinical platforms today have a sensitivity of only about 1%, owing largely to the intrinsic error rate of polymerase enzymes required for adequate sample preparation. Using enzymes with proofreading capability can incrementally improve this sensitivity, as can computational methods, but the best options for drastic improvement in sensitivity of NGS for MRD likely involve using sequencing variations, such as error-corrected NGS [42–44] or duplex sequencing [45, 46]. These techniques may be able to reach sensitivities of 10^–6^ in some cases. Efforts to use peripheral blood (e.g., circulating cell-free DNA) as a relatively non-invasive source for NGS analysis and MRD determination are also ongoing, with some previous reports suggesting that this approach is feasible in AML [47, 48].

## MRD as a prognostic biomarker

It is logical to hypothesize that the presence of any detectable residual cancer cells, i.e., MRD, that persist or recur after treatment with chemotherapy would generally portend relapse. For nearly thirty years, dozens of studies have supported this hypothesis [8–14]. More recently, a meta-analysis of 81 trials with over 11,000 patients found strong associations between MRD negativity and superior disease-free survival (DFS; 5-year DFS 64% vs. 25% for patients with MRD; HR 0.37 [95% CI 0.59, 0.70]) and OS (5-year OS 68% vs. 34%; HR 0.36 [95% CI 0.33, 0.39]). The strong adverse prognostic effect of MRD positivity was observed across patient or disease characteristics, including age (i.e., children vs. adults), timing of MRD assessment, AML subtype, or specimen source (i.e., BM vs. peripheral blood [PB]), underscoring the critical value of MRD in AML across clinical contexts [14]. Here, we summarize the literature regarding MRD as prognostic biomarker in AML.

### MRD assessment by RT-qPCR

RT-qPCR has been predominantly used in two major subtypes of AML—those with *NPM1* mutations and those with core binding factor (CBF) AML. Below, we have stratified the role of RT-qPCR in MRD assessment according to these disease subtypes.

#### *NPM1*-mutant AML

Some of the most robust and consistent evidence for the prognostic role of MRD comes from studies of PCR-based MRD assessment in *NPM1-*mutant AML. Tracking the presence of this mutation is an ideal strategy for MRD detection because of its stability throughout disease and its specificity to AML [49–51]. Mutations in *NPM1* are not observed in CHIP, and appear to be founding genetic events in AML [52]. This is highlighted by the fact that nearly 95% of relapses of *NPM1*-mutant AML retain the *NPM1* mutation at the time of relapse, with only rare relapses being *NPM1* wild type clones [11, 53, 54]. In European countries in particular, RT-qPCR is the standard method for measuring MRD in this setting, as MRD detection by this method has been shown to predictably correlate with relapse and long-term survival for over 15 years, and is well-standardized through efforts of the ELN and others [7, 55]. However, in the United States and many other countries, RT-qPCR for mutant *NPM1* is not widely available nor is it standardized, thus limiting its use in these regions.

Detection of mutant *NPM1* by RT-qPCR in PB or BM after two cycles of induction chemotherapy has been shown to be strongly predictive of relapse and decreased survival [11, 56]. In one study of younger patients with newly diagnosed *NPM1*-mutated AML, cumulative incidence of relapse (CIR) for patients with detectable MRD after double induction was 53.0% at 4 years versus only 6.5% for those with undetectable MRD (*P* < 0.001) [56]. These findings were echoed in a larger study of 346 patients, where the 3-year CIR rate was 82% in patients with MRD after 2 cycles of intensive chemotherapy versus 30% in patients without MRD (*P* < 0.001) [11]. In line with these findings, current ELN recommendations for monitoring of mutant *NPM1* include MRD assessment by RT-qPCR, at a minimum, after two cycles of chemotherapy, then at 3-month intervals for at least 2 years after the end of treatment [23].

MRD positivity (particularly > 1% mutant *NPM1* by RT-qPCR) before hematopoietic stem cell transplant (HSCT) is associated with poor outcomes [57, 58]; however, HSCT still has substantial therapeutic benefit for persistent MRD positivity [59, 60]. Even in patients with detectable MRD pre-transplant, low levels (i.e., < 200 copies/10^5^ *ABL1* in PB and < 1000 copies/10^5^ *ABL1* in BM) have been associated with a low-risk of relapse post-transplant, as long as no concomitant *FLT3* mutation is present [58]. Detection of mutant *NPM1* transcripts post-HSCT has also been shown to be a reliable indicator of impending relapse [61].

#### CBF AML

The fusion transcripts in CBF AML (*RUNX1-RUNX1T1* or *CBFB-MYH11*) resulting from t(8;21) or inv(16)/t(16;16), respectively, are readily detected by RT-qPCR at a sensitivity ranging from 10^–4^ to 10^–6^ [15]. Controversy remains regarding the optimal timing of MRD assessment for therapeutic decision-making in CBF AML and the threshold levels of detectable fusion transcripts that are truly predictive of relapse. In this context, it is imperative to note that a minority of patients in long-term remission may have stably low levels of detectable fusion transcripts [62–64].

While a negative result is the most desirable PCR outcome that puts both patient and provider at ease, several studies have evaluated threshold values or trends of PCR positivity that provide optimal predictive utility [62, 65–69]. Earlier in treatment, slightly higher PCR ratios seem to be allowable for a favorable prognosis; as treatment continues, lower cutoff values of PCR ratios associated with favorable outcomes are required. At the higher end of this cutoff, for example, one study found favorable continuous CR rates with PCR values < 0.5% post-induction (76% vs. 36% at 2 years; *P* = 0.01) and at < 0.1% after first consolidation (74% vs. 40% at 2 years; *P* = 0.02) [65]. On the lower end, another study found < 0.1% post-induction and < 0.01% post-consolidation to be the most prognostic cutoffs [66].

The large prospective UK MRD AML15 trial determined cutoff values that could predict 100% relapse risk in both t(8;21) or inv(16) [67]. In patients with inv(16), > 10 copies of *CBFB-SMMHC* in PB any time after first consolidation or > 50 copies in BM after second consolidation were associated with a 5-year relapse risk of 100%. Similarly, in patients with t(8;21), > 500 copies of *RUNX1-RUNX1T1* in BM any time after first consolidation or > 100 copies in PB after second consolidation were associated with universal relapse. Morphologic relapse predictably occurred within 3 months of these cutoff values for patients with inv(16) or within 4.5 months for patients with t(8;21). These findings translated into statistically significant differences in OS in the t(8;21) group, with 5-year OS 94% versus 57% for patients with > 500 fusion transcripts in BM (*P* = 0.001). However, no OS difference was found in the inv(16) group, perhaps owing to superior salvage treatment options for these patients.

### MRD assessment by MFC

MFC measurement of MRD yields prognostic information and can guide treatment decisions for a large portion of patients with AML [15]. A comparative study between MFC-assessed MRD status and clinical response (i.e., CR, CR with incomplete platelet recovery [CRp] or CR without blood count recovery [CRi]), found a significant correlation between MRD and response status after induction [70]. MRD was more often detectable in patients with CRi or CRp than in those with CR (60.9% vs. 54.2% vs. 19.0%, respectively; *P* < 0.01). Patients with CRi or CRp had higher levels of MRD than those observed in patients in CR. Importantly, MRD response and hematologic response were both independently prognostic for outcomes. These findings provided some of the most robust evidence that supported the development of the response criterion of CR_MRD-_ by the ELN [23].

The AML17 trial evaluated the prognostic value of MFC-measured MRD in 2450 mostly younger patients (< 60 years) with standard risk, *NPM1* wild type AML. After one cycle of induction chemotherapy, patients without MRD had better OS (70% vs. 51% at 5 years; *P* < 0.001) than those with detectable MRD. Interestingly, patients with MRD had 5-year OS akin to that for patients in only partial remission (51% vs. 46%). MRD ≥ 1% after two cycles of therapy was associated with an 89% relapse risk and shortened OS (33% vs. 63%, *P* = 0.003) as compared to those with lower levels or undetectable MRD. Importantly, allogeneic-HSCT (allo-HSCT) appeared to be more beneficial in patients with persistent MRD than those who were MRD negative, suggesting that allo-HSCT should perhaps be preferentially recommended to patients with standard risk *NPM1* wild type AML with positive MRD after induction [9].

The observation that pre-transplant MRD portends inferior post-HSCT outcomes has been shown in several studies, many of which have utilized MFC to detect MRD [71–74]. In a meta-analysis evaluating the impact of MRD prior to allo-HSCT, MRD positivity was associated with shortened leukemia-free survival (LFS) and OS, and increased CIR [75]. These relationships were evident regardless of whether RT-qPCR or MFC was used for MRD assessment, though studies using MFC were less uniform in the resulting survival estimates, likely attributable to the lack of standardization in this platform. In a particularly illustrative study, 3-year OS in patients with even very low levels of pre-HSCT MRD was remarkably similar to patients transplanted with active disease (26% vs. 23%), highlighting the adverse prognostic impact of pre-HSCT MRD [74]. The outcomes of patients with active disease at the time of allo-HSCT or who had detectable pre-HSCT MRD were far inferior to those of patients in an MRD-negative state pre-HSCT, where the latter had a relatively favorable 3-year OS of 73%.

Detectable MRD post-HSCT is also highly prognostic of clinical outcomes [73, 76]. Post-HSCT MRD strongly predicts relapse incidence, particularly when observed within 30 days after transplant, and was associated with a 1-year relapse incidence of 78% in one study [76]. Another study found that patients with MRD persisting after transplant had a 3-year risk of relapse of 81% and a 3-year OS of only 17%. In comparison, patients without detectable post-HSCT MRD had substantially improved 3-year relapse and OS rates of 31% and 67%, respectively [73].

### MRD assessment by targeted NGS

Leukemia-associated mutations are common in AML and can be readily identified with NGS [39]. As this platform has become more widely available, NGS-based MRD assessment has emerged as having prognostic utility [13, 37, 42, 77, 78]. Prior to the use of targeted NGS panels, Klco et *al.* used whole-genome or exon sequencing to evaluate mutational burden in 50 patients one month after induction chemotherapy. Patients without persistently detectable mutations or with detectable mutations but VAFs ≤ 2.5% had significantly improved event-free survival (EFS; median 17.9 months vs. 6.0 months; *P* < 0.001) and OS (median 44.2 months vs. 10.5 months; *P* = 0.004) compared to those with persistent mutations with VAF > 2.5% [77]. Using targeted NGS spanning 295 genes, a study of 131 patients found better CIR and OS in patients with undetectable mutations compared with those with residual mutations (any detectable VAF) at 30 days post-induction (2-year CIR 24% vs. 46%; *P* = 0.03; 2-year OS 77% vs. 60%; *P* = 0.03). Removing CHIP-associated “DTA” (*DNMT3A, TET2,* or *ASXL1*) mutations from analysis slightly expanded the difference in 2-year OS (81.8% vs. 62.9%; *P* = 0.03) [37].

Similarly, in the largest MRD study of targeted NGS in AML (482 patients, 54 genes), presence of any non-DTA leukemia-associated mutation at time of CR was associated with a higher relapse rate at 4 years (55.4% vs. 31.9% if no detectable mutation; *P* < 0.001), independent of VAF [13]. Persistent non-DTA mutations also conferred shortened OS (41.9% vs. 66.1% at 4 years; *P* = 0.001) [13]. Persistence of only DTA mutations in CR did not have prognostic importance. Targeted NGS and MFC had similar independent prognostic utility in the detection of MRD and provided additive prognostic value. The 4-year relapse rate was 73.3% in patients with MRD detectable by both platforms, whereas the 4-year relapse rate was 26.7% for patients without detectable MRD by either platform. Patients with MRD detectable by only one of the assays had an intermediate prognosis, with 4-year relapse rates of 52.3% and 49.8% in patients with MRD detected only by NGS or only by MFC, respectively. It is notable that despite combining two different MRD platforms, over a quarter of patients who were MRD negative by both NGS and MFC still relapsed. This finding is perhaps in part due to the limitations of targeted sequencing including a small gene list and, importantly, highlights the lack of sensitivity and imperfect nature of our currently available MRD assays.

MRD detected by NGS prior to HSCT is also a negative prognostic indicator. Using a targeted panel of 24 genes subjected to a more sensitive, error-corrected NGS, one study found pre-HSCT MRD to be associated with 5-year CIR of 66% (versus 17% in MRD-negative cases; *P* < 0.001) and 5-year OS of 41% (versus 78% in MRD-negative cases; *P* = 0.002) [42]. These findings have been echoed by others, even with less sensitive NGS methods [79]. While its role after HSCT is less clear, NGS detection of MRD may also be of prognostic value, particularly when combined with MFC or when MRD is present prior to transplant [78, 80, 81].

### Integrating MRD information across assays

Because qRT-PCR/NGS and MFC identify MRD in fundamentally distinct manners—genomic versus phenotypic aberrations—these methods may be complementary in the assessment of MRD. This complementarity has been shown, for example, in the context of MFC and NGS, where the information provided by each assay adds important prognostic information [13]. Additionally, because each MRD platform differs in its advantages and disadvantages (Table 1), the integration of more than one method of MRD assessment can provide a more complete picture of disease status in a given patient. For example, both genomic and phenotypic clonal evolution are well-described phenomena in AML, and there is potential for resistant subclones to expand (or new clones to emerge) due to selective pressure from treatment [4]. This potential for clonal evolution and/or immunophenotype shifts makes MRD monitoring using multiple platforms particularly important, as it is possible that clinically significant MRD might be missed when only one assay is used. Given the established prognostic impact of MRD across different assays [14], we routinely assess MFC- and NGS-based MRD concomitantly (and also include PCR when there is an appropriate target).

### Prognostic value of MRD in selected patient populations

#### MRD in older adults with AML receiving lower-intensity therapies

While most studies evaluating the prognostic utility of MRD are done in the context of intensive chemotherapy, MRD measured by MFC retains its discrimination for relapse risk when applied to older adults (> 60 years) treated with lower-intensity frontline regimens of hypomethylating agents (HMAs; azacitidine or decitabine). In one study, patients with MRD at time of CR/CRi/CRp had inferior 2-year CIR compared with patients harboring no MRD (84% vs. 43%; *P* < 0.001). However, these disparate relapse rates did not translate to differences in relapse-free survival (RFS) or OS, likely due to competing risk of death in this older, frail population [82].

A recent study evaluated the prognostic utility of MRD evaluated by MFC in older patients with AML after venetoclax in combination with 10-day decitabine [83]. Overall, 54% of responders achieved MRD negativity at some point over the course of therapy, with notably higher rates of MRD negativity achieved in patients with intermediate-risk cytogenetics (67%) compared to those with adverse-risk cytogenetics (33%). Compared with MRD-positive patients, those who achieved MRD negativity within 2 cycles of therapy had significantly longer RFS (median not reached vs. 5.2 months; *P* = 0.004) and OS (25.1 months vs. 7.1 months; *P* < 0.0001). Similar magnitudes of benefit were observed with MRD-negativity after 1 or 4 cycles of therapy. Thus, MRD appears to remain a useful prognostic tool after lower-intensity induction therapy for older patients with AML [83].

#### MRD in relapsed/refractory AML

There are relatively scant data available regarding the impact of MRD in relapsed/refractory AML. In one study of adults with relapsed/refractory AML undergoing first salvage therapy, achievement of MRD negativity by MFC was associated with significantly lower CIR, longer RFS, and a trend toward superior OS [84]. Similar to what has been shown in newly diagnosed AML [70], full hematologic recovery (i.e., CR) and MRD negativity were both independent prognostic factors for CIR and RFS, thus supporting the benefit of achieving CR_MRD-_ even in the relapsed/refractory setting. The superior outcomes of patients who achieved MRD negativity in second CR were largely driven by a lower risk of early relapse, allowing these patients more often to be successfully bridged to potentially curative HSCT. Interestingly, among patients who proceeded to HSCT, the MRD response to salvage therapy did not impact post-HSCT outcomes. This suggests that while achievement of MRD negativity in the relapsed/refractory setting is ideal, undergoing HSCT is ultimately more important than depth of response [84]. These findings are in stark contrast to many studies in the frontline setting where MRD response prior to HSCT is highly prognostic for post-HSCT outcomes [71–75].

## MRD-directed therapeutic approaches

### MRD-guided HSCT

Optimizing post-remission therapy for patients with AML remains a major clinical challenge. Allo-HSCT is a potentially curative option for many patients, but must be pursued sapiently given its substantial risk of potential toxicity [7, 85]. Given its prognostic value, MRD may serve as a useful therapeutic decision-making tool to identify which patients may be best suited for HSCT.

In *NPM1-*mutant disease, younger patients (18–60 years) with < 4 log reduction in PB MRD by RT-qPCR post-induction who underwent allo-HSCT in first CR had significantly better DFS (hazard ratio [HR], 0.25; *P* = 0.047) and OS (HR 0.25; *P* = 0.030) compared to those who did not undergo allo-HSCT [59]. A critical observation was made that HSCT conferred no significant benefit to patients with *NPM1-*mutant AML who did not have detectable MRD or had > 4 log reduction in MRD prior to allo-HSCT. These data support the consideration of MRD response in guiding the decision for consolidative HSCT in patients with *NPM1*-mutated AML and suggest that allo-HSCT in first remission might be preferentially reserved for patients with suboptimal MRD response.

The utility of MRD to inform decisions regarding HSCT in first remission has also been shown in CBF AML. The AML05 trial used MRD to risk-stratify 116 patients with t(8;21) disease and guide subsequent therapy [86]. “High-risk” disease was defined as achievement of a < 3-log reduction in RT-qPCR transcripts after second consolidation or re-appearance of fusion transcripts within 6 months of a previously undetectable result. Among high-risk patients, allo-HSCT was associated with significantly lower 5-year CIR than consolidation chemotherapy (22.1% vs. 78.9%; *P* < 0.0001) and improved 5-year OS (71.6% vs. 26.7%; *P* = 0.007). In contrast, in patients with low-risk disease (i.e., ≥ 3-log PCR reduction from baseline after second consolidation that was sustained for 6 months), allo-HSCT did not decrease relapse risk and was associated with worse OS compared to chemotherapy, most likely due to introduction of transplant-related complications and mortality in these low-risk patients. Similar trials in inv(16) disease would be worthwhile to determine the role of MRD-directed HSCT in this subset.

The AML17 trial investigated the impact of MRD status in standard-risk, *NPM1* wild type AML [9]. The 5-year OS for patients without MRD by MFC after 2 cycles of chemotherapy was 88% (versus 35% for MRD positive cases; *P* < 0.001) when these data were censored for HSCT. Importantly, there was a strong trend suggesting an interaction between MRD status and benefit from allo-HSCT in first remission. Although the subgroup analyses were not statistically significant, MRD positive patients trended toward better OS if they underwent HSCT, whereas those who were MRD negative trended toward inferior OS if they underwent HSCT. Only 44 patients underwent allo-HSCT in this subgroup analysis, lending caution to the interpretation of these findings. Nevertheless, this study suggests that MRD status may be a useful consideration for pursuing or deferring transplant in patients with standard-risk, *NPM1* wild type AML.

The GIMEMA AML1310 trial prospectively used MRD to guide HSCT strategy in young adults with newly diagnosed AML [87]. In this study, patients with intermediate-risk cytogenetic/molecular features and with detectable MRD after consolidation underwent allo-HSCT and those without detectable MRD underwent autologous HSCT (auto-HSCT). Interestingly, among these two groups of intermediate-risk patients, there was no statistically significant difference in either 2-year OS (79% in MRD-negative vs. 70% in MRD-positive; *P* = 0.713) or DFS (61% in MRD-negative vs. 67% in MRD-positive; *P* = 0.773). These findings suggest that an MRD-directed selection of HSCT consolidation may overcome the negative prognosis of MRD positivity in intermediate-risk patients.

In our practice, we routinely refer patients with MRD positivity after first induction for HSCT, meanwhile continuing therapy with the goal of minimizing—and, ideally, eliminating—MRD prior to HSCT as a suitable donor is being identified. For patients who are standard/intermediate-risk and who achieve MRD negativity after induction, the role of HSCT is less clear. In these cases, the decision to proceed to HSCT should be individualized and should be based on the patient’s expected risk of HSCT-related morbidity and mortality, as well as personal preferences after an informed discussion of risks and benefits.

Once plans are made to proceed with HSCT, decisions regarding the optimal donor source or type of conditioning regimen may be influenced by pre-HSCT MRD status. Many patients lack a matched related donor, raising the question of whether alternative stem cell sources can provide benefit to these individuals. Indeed, transplants using cord-blood derived stem cells or haploidentical sources have shown acceptable outcomes, particularly for patients with MRD [88–90]. Recent data also suggest that MRD status can reasonably be used to guide pre-HSCT conditioning intensity [38]. In a study of adults undergoing allo-HSCT, patients were randomized to receive myeloablative conditioning (MAC) or reduced-intensity conditioning (RIC). MRD was subsequently assessed from preconditioning samples of 190 patients using a targeted panel of 13 genes and error-corrected NGS. Of patients who relapsed post-transplant, 71% were MRD positive, consistent with other studies supporting pre-HSCT MRD as a negative prognostic indicator [42, 74, 79]. Conditioning regimen did not appear to influence OS in patients without detectable residual mutations pre-HSCT (56% in MAC group vs. 63% in RIC group at 3 years; *P* = 0.96). However, patients with detectable MRD who were randomly assigned to RIC had a significantly higher CIR compared with those assigned to MAC (1-year CIR 58% vs. 14%; *P* < 0.001) and worse OS (3-year OS 43% vs. 61%; *P* = 0.02). These trends became even more pronounced when DTA mutations were excluded from analysis, with patients receiving RIC experiencing higher CIR and shorter survival compared to MAC (3-year CIR 72% vs. 15%; *P* < 0.001; 3-year OS 34% vs. 59%; *P* = 0.01). Taken together, this supports the consideration of intensive conditioning regimens for patients with MRD undergoing allo-HSCT, whenever possible.

### Non-HSCT MRD-directed therapies

Once identified, understanding and combating the resilient cells composing MRD is critical to prevent relapse and improve patient outcomes. After exposure to chemotherapy, AML cells enter a senescence-like state that confers protection from the cytotoxic effects of chemotherapy, allowing these cells to evade death and later repopulate, causing relapsed disease [91]. In addition, LSCs (a small population of immature, drug-resistant cells capable of repopulating bulk AML disease) may also be present, though perhaps not enriched, after treatment [92, 93]. Several studies are evaluating MRD-directed approaches with the goal of eradicating MRD in patients with persistent or recurrent MRD after conventional therapy. This approach is informed by the success of the CD3-CD19 bispecific engaging antibody blinatumomab in the treatment of MRD-positive B-cell acute lymphoblastic leukemia (ALL), which is FDA approved for this indication [94]. Ongoing studies of MRD-directed therapy in AML are shown in Table 2.

**Table 2:** Select ongoing trials for which patients with MRD are eligible

Trial number	Phase	Patient population	Timing of MRD positivity	Intervention	Primary Outcome	MRD platform
NCT04809181	2	AML/MDS; < 65 years	After allo-HSCT	Azacitidine + venetoclax	RFS	MFC or RT-qPCR
NCT04689815	2	*NPM1*-mutant AML	After consolidation or post-HSCT	Azacitidine + arsenic trioxide	Rate of *NPM1* MRD negativity	RT-qPCR
NCT04712942	2	AML/MDS	After consolidation or post-HSCT	Azacitidine +/− pevonedistat	MRD status	MFC or RT-qPCR
NCT04086264	1/2	CD123+ AML	After frontline treatment	IMGN632	Antileukemia activity, MRD levels	MFC
NCT03769532	2	*NPM1-*mutant AML*;* post-HSCT excluded	After chemotherapy	Azacitidine + Pembrolizumab	EFS	RT-qPCR
NCT04541277	2	AML with PDL1 expression; > 60 years or unfit for intensive chemotherapy; post-HSCT excluded	Not specified	Tislelizumab + HMA +/− CAG	ORR	Not specified
NCT02684162	2	AML/MDS	After allo-HSCT	Guadecitabine + DLI	CRR, RFS	MFC, RT-qPCR, NGS
NCT02789254	1/2	AML with FLT3 expression; post-HSCT excluded	After any therapy	FLYSYN	Safety/tolerability	NGS or RT-qPCR
NCT02520427	1	AML/MDS	Not specified	AMG330	Safety/tolerability	Not specified
NCT02275533	2	AML in first remission	After induction or consolidation	Nivolumab	PFS	Not specified
NCT04623216	1/2	AML in remission after HSCT	After allo-HSCT	Sabatolimab +/− Azacitidine	Safety/tolerability, CRR	Not specified
NCT02126553	2	AML ≤ 55 years	After induction	Lenalidomide	RFS	MFC, RT-qPCR
NCT02770820	1/2	Elevated WT1 expression, HLA-A*02.01 genotype	After induction or consolidation	WT1 directed allo-CD8 T cells	Safety/tolerability	MFC, RT-qPCR
NCT04209712	1	AML (includes children)	After 2 courses of chemotherapy	allo-NK cells	MRD response	MFC
NCT04632316	1/2	AML/MDS	After consolidation	oNKord	Safety/tolerability, MRD response	MFC
NCT04284228	1/2	AML/MDS; HLA-A2.01 expression	After allo-HSCT	NEXI-001	Safety/tolerability, PFS, ORR, OS	MFC or NGS
NCT03697707	2	AML in first remission	After induction or consolidation	DCP-001	MRD response	MFC
NCT04580121	1	HLA-A*02 genotype	Any time	RO7283420	Safety/tolerability	MFC
NCT03737955	2	CD33+ AML/MDS/MPN; (includes children)	Any time	Gemtuzumab ozogamicin	MRD response	MFC or RT-qPCR
NCT04526288	2	AML/MDS	After chemotherapy	allo-HSCT +/− preceding CPX351	OS	MFC, RT-qPCR, NGS, FISH, or cytogenetics
NCT03537599	1/2	AML	After allo-HSCT	Daratumumab + DLI	Safety/tolerability, ORR	MFC, NGS, or cytogenetics
NCT03793517	2/3	Acute leukemias with MLL-r, TLS-ERG, or SIL-TAL1; < 55 years	pre-HSCT	Decitabine + mBu/Cy + allo-HSCT	CIR	Not specified
NCT03728335	1	*IDH2*-mutated AML	After allo-HSCT	Enasidenib	Safety/tolerability	MFC, NGS
NCT04326764	3	AML/MDS	pre-HSCT	Panabinostat	OS	MFC or RT-qPCR

*MRD* measurable residual disease, *AML* acute myeloid leukemia, *MDS* myelodysplastic syndrome, *allo-HSCT* allogeneic hematopoietic stem cell transplant, *RFS* remission-free survival, *MFC* multiparameter flow cytometry, *RT-qPCR* reverse transcription-quantitative polymerase chain reaction, *EFS* event-free survival, *HMA* hypomethylating agent, *CAG* cytarabine, idarubicin, granulocyte colony-stimulating factor, *ORR* overall response rate, *DLI* donor lymphocyte infusion, *CRR* complete response rate, *NGS* next-generation sequencing, *PFS* progression-free survival, *allo-NK cells* allogeneic natural killer cells, *OS* overall survival, *mBu/Cy* modified busulfan and cyclophosphamide

#### HMAs

HMAs have been used in various studies to treat persistent or recurrent MRD in AML. In CBF AML, one study suggested that HMA therapy may be effective in patients with low levels of MRD by RT-qPCR (0.01–0.05%) after consolidation. This small study (*n* = 23) found that 6/6 patients without MRD (RT-qPCR < 0.01%) treated with HMAs remained MRD negative at follow-up (median follow-up 11.3 months, range 2.9–67.7 months). Additionally, 12/17 patients with residual MRD at start of HMA therapy remained in remission at follow-up, with 11/12 experiencing decreased RT-qPCR values within 1–2 cycles of HMA therapy [95]. Randomized studies with longer follow-up are needed to interrogate the utility of maintenance therapy, with HMA or with other novel agents, in this patient population. Similarly, in *NPM1-*mutant disease, a small study of 10 patients suggested that azacitidine may be able to prevent relapse in some patients with persistent or increasing RT-qPCR detectable MRD (≥ 1%). At 10-month follow-up, 7/10 patients remained in CR, all with decreasing RT-qPCR values ≥ 1 log [96]. Randomized studies with longer follow-up are needed to fully assess the utility of MRD-directed therapy with HMA-based regimens in these patient populations.

The RELAZA-2 trial is the largest study to date to evaluate the utility of HMA therapy in patients with MRD-positive AML [97]. In this study, 53 patients with AML or MDS in CR with positive MRD by either a RT-qPCR-trackable molecular aberration (≥ 1% mutant *NPM1, RUNX1-RUNX1T1, CBF-MYH11,* or *DEK-NUP214*) or decreased donor CD34 chimerism ≤ 80% by MFC (in patients who were post-HSCT) were treated with azacitidine. After azacitidine therapy, 19 of 53 patients (36%) converted to an MRD-negative state. The 1-year RFS and OS rates after start of azacitidine were 46% and 75%, respectively. Importantly, conversion to an MRD-negative state was associated with better outcomes, with these patients having 1-year RFS and OS rates of 88% and 91%, respectively. Although over half of patients still relapsed within 1 year, azacitidine appeared to have delayed this substantially, with a median time to relapse of 422 days in patients treated with azacitidine (versus 61 days observed in historical controls).

The QUAZAR AML-001 study of CC-486 (oral azacitidine) provides further evidence for the use of HMAs as maintenance therapy for AML, including in patients with positive MRD [98]. In this randomized, phase III study, older patients (≥ 55 years) who achieved remission after intensive chemotherapy, but who were deemed ineligible for HSCT, were randomized to CC-486 or placebo as maintenance therapy. In the overall study cohort, patients receiving CC-486 maintenance had improved OS (median 24.7 months vs. 14.8 months with placebo; *P* < 0.001) and RFS (median 10.2 months vs. 4.8 months with placebo; *P* < 0.001). Interestingly, CC-486 conferred an OS and RFS benefit regardless of MRD status at time of randomization [99]. Patients with MRD at time of randomization to CC-486 had improved OS compared to those in the placebo group (median 14.6 months vs. 10.4 months; HR 0.69 [95% CI 0.51, 0.93]) and RFS (7.1 months vs. 2.7 months; HR 0.58 [95% CI 0.43, 0.78]). This benefit was still evident in patients without MRD, as median OS with CC-486 was 30.1 months versus 24.3 months with placebo (HR 0.81 [95% CI 0.59, 1.12]); and median RFS 13.4 months versus 7.8 months (HR 0.71 [95% CI 0.52, 0.98]). The benefit of CC-486 in MRD-positive patients was at least partially driven by its increased MRD conversion rate compared to placebo (37% vs. 19%, respectively). CC-486 is now FDA-approved as maintenance therapy in patients with AML in first CR who are unable to complete intensive curative therapy. Even with this MRD-directed therapy, the 5-year OS for these patients was only ~ 30%, indicating that HMA therapy alone is largely insufficient for cure, and other agents are likely to be necessary to improve long-term outcomes.

#### Venetoclax-based combinations

Venetoclax-based regimens may be useful for both persistent MRD and re-emerging MRD when combined with low-dose cytarabine or azacitidine. In a small study of patients with mutant *NPM1*, all five patients with persistent MRD after consolidation converted to an MRD-negative state after 1–4 cycles of treatment with either azacitidine or low-dose cytarabine plus venetoclax [100]. In the cohort of patients with rising or re-emerging MRD, venetoclax combination therapy with low-dose cytarabine or azacitidine induced an MRD-negative state in 6 out of 7 patients within two cycles. Remarkably, with a median follow-up of 10.8 months, all patients who became MRD-negative remained in CR. These encouraging findings support further study of these combinations in larger trials.

NCT04062266 is a single-arm study currently underway to evaluate the combination of azacitidine with venetoclax on survival outcomes in patients with high-risk AML in CR who are ineligible for HSCT, including those with persistent or recurrent MRD. This study will assess MRD response as a secondary outcome. Interestingly, NCT03466294 is also ongoing to evaluate venetoclax with azacitidine as induction therapy in elderly patients with AML. In this study, MRD negativity is a secondary endpoint, and will be used to de-escalate therapy by removing azacitidine and transition patients to a venetoclax monotherapy maintenance regimen. Given the synergy seen with HMAs and venetoclax and the recent approval of the orally available formulation of azacitidine, CC-486, combination studies of CC-486 and venetoclax would be worthwhile. In addition, treating patients with completely oral regimens may improve compliance, quality of life, and potentially long-term outcomes.

#### FLT3 inhibitors

The SORMAIN trial evaluated 24 months of post-allo-HSCT sorafenib maintenance versus placebo in 83 patients with *FLT3*-internal tandem duplication (*FLT3-*ITD) AML [101]. Overall, sorafenib improved RFS (85.0% vs. 53.3% at 2 years; *P* = 0.002) and OS (90.5% vs. 66.2% estimated at 2 years; *P* = 0.007). Patients with undetectable MRD prior to allo-HSCT derived the most benefit from sorafenib, as no sorafenib-treated patients relapsed or died (*n* = 9) compared with 5/12 patients who received placebo (*P* = 0.028). After HSCT, patients with MRD had improved RFS with sorafenib compared with placebo (67% vs. 20% at 2 years; *P* = 0.015), whereas the benefit of sorafenib in patients who were MRD negative post-HSCT was less clear.

In a similar trial evaluating sorafenib maintenance in the first six months after allo-HSCT in 202 patients with *FLT3-*ITD AML, sorafenib conferred improvements in CIR and prolonged LFS and OS (1-year CIR 7.0% vs. 24.5% with placebo, *P* = 0.001; 2-year LFS 78.9% vs. 56.6%, *P* < 0.001; and 2-year OS 82.1% vs. 68.0%, *P* = 0.012) [102]. Patients who were MRD negative post-HSCT had a CIR of 9.8% with sorafenib (versus 26.3% with placebo) at two years (HR 0.28, [95% CI 0.13, 0.62]). Meanwhile, the few patients with post-HSCT MRD had a CIR of 33.3% with sorafenib (*n* = 9) versus 77.3% with placebo (*n* = 11; HR 0.25, [95% CI 0.06, 0.94]). While patients with MRD at any time had inferior outcomes compared to patients without MRD, sorafenib was associated with a lower incidence of relapse regardless of MRD status.

Given the broad kinome of sorafenib, which inhibits several non-FLT3 targets, future studies evaluating TKIs with more specificity for FLT3 would be of great interest to determine whether these clinically beneficial effects are due to on-target FLT3 inhibition or more broad inhibition of leukemic and immunologic kinase activity. Studies using other TKIs in the post-HSCT setting, including midostaurin, recently concluded (NCT04027309) [103], and a study including gilteritinib is underway (NCT02997202).

### Practical management of MRD-positive AML

To date, the best data support the use of allo-HSCT for patients with AML and persistent or recurrent MRD-positivity. A myeloablative, rather than reduced-intensity, conditioning regimen should be used whenever possible, as this strategy may overcome the adverse prognosis of MRD [38]. The role of MRD-directed therapies in patients who are not suitable candidates for HSCT is less clear. Enrollment in an MRD-directed clinical trial is always preferred. However, for patients who received intensive induction, CC-486 has showed a survival benefit and is a reasonable option [98]. Although there are limited data to support the use of HMA plus venetoclax in this context, given the established superiority of HMA plus venetoclax (versus HMA alone) in the frontline setting [104], our own practice is to use this doublet regimen for such patients, rather than an HMA alone. For patients who received frontline therapy with an HMA plus venetoclax and who have persistent MRD after 4–6 cycles along with MRD values that fail to quantitatively decline on sequential assessments, a clinical trial should be strongly considered.

## MRD as a surrogate endpoint

Because MRD status can be assessed as early as after the first induction cycle, using MRD as a surrogate endpoint in clinical trial design could dramatically expedite drug approval, rather than waiting years for long-term OS data to mature. Use of MRD as a surrogate endpoint would also reduce trial cost, as it could realistically shorten the required time to execute a large clinical trial. To consider MRD as a surrogate endpoint for OS, it is first necessary to demonstrate strong evidence for the association of MRD and OS. This association has consistently been shown across dozens of large AML studies and was recently quantified in a meta-analysis of 81 different studies [14]. In this meta-analysis, achievement of MRD negativity was associated with doubling of the OS (5-year OS 68% vs. 34% in those who were MRD positive; HR 0.36 [95% CI 0.33, 0.39]). However, for regulatory approval of MRD as a surrogate endpoint, it is important to also show consistent data from prospective clinical trials showing that treatment effects on MRD status correlate with similar changes in OS. Thus, it is imperative that MRD status should be included as a pre-defined endpoint in AML therapeutic trials. Such an endeavor also necessitates standardization of assays used to detect MRD across participating laboratories. This information will support the use of MRD as a surrogate endpoint for regulatory approval and will also allow for the possible approval of MRD-directed therapies in AML, like what was achieved by blinatumomab for ALL. Clinical trials using MRD status as a primary endpoint (excluding those evaluating MRD-directed therapies for patients with MRD-positive disease) are outlined in Table 3.

**Table 3:** Select ongoing trials using MRD as a primary endpoint

Trial number	Phase	Patient population	Intervention	Primary outcomes	MRD platform
NCT04168502	3	*FLT3* wild type AML ≤ 60 years	Chemotherapy + GO induction and consolidation; Glasdegib as post-HSCT maintenance	MRD negativity, DFS	Not specified
NCT04093505	3	AML ≥ 60 years	Chemotherapy + GO in induction (dosed day 1 vs. days 1, 4, 7); Glasdegib (versus placebo) in consolidation and maintenance	MRD negativity	MFC
NCT04284787	2	AML > 60 years	Azacitidine + Venetoclax +/− Pembrolizumab	MRD negative CR	MFC
NCT04214249	2	*FLT3* wild type	Cytarabine + Idarubicine +/− Pembrolizumab	MRD negative CR	MFC
NCT03150004	2	Relapsed/refractory or secondary AML	CLAG-M	MRD negative CR	Not specified
NCT03549351		Any enrolled in specified interventional prospective randomized trials	Observational study	Correlation between MRD and OS	MFC

Trials using MRD as an independent primary outcome, except those trials that specifically include patients in MRD-positive remission (as shown in Table 2). *GO* gemtuzumab oligomycin, *HSCT* hematopoietic stem cell transplant, *MRD* measurable residual disease, *DFS* disease-free survival, *MFC* multiparameter flow cytometry, *MDS* myelodysplastic syndrome, *allo-NK cells* allogeneic natural killer cells, *UCB* umbilical cord blood, *IL-2* interleukin-2, *RT-qPCR* reverse transcription-quantitative polymerase chain reaction, *G-CSF* granulocyte colony stimulating factor, *CR* complete response, *CLAG-M* cladribine, cytarabine, granulocyte colony stimulating factor, mitoxantrone, *OS* overall survival, *CR*_*MRD-*_ complete response with no measurable residual disease, *NRM* non-relapse mortality, *CRR* complete response rate, *DLI* donor lymphocyte infusion, *CLAM* clofarabine, cytarabine, mitoxantrone

## Conclusion

The body of evidence supporting the role of MRD as a prognostic indicator in AML is abundant. Further efforts to standardize testing and interpretation including sample source and timing of MRD assessment will further strengthen the clinical utility of MRD. In addition, we must strive to comprehend how MRD can guide therapy in AML and whether certain subtypes of disease may warrant differential treatment depending on MRD status. To achieve anything resembling a cure in AML, we must tackle MRD in an informed manner—whether that means prolonged maintenance therapy, HSCT, a change of therapy with incorporation of novel agents, etc. At present, the best available evidence supports the consideration of HSCT and/or HMAs for patients with persistent or recurrent MRD. However, the outcomes for many patients remain poor even with these approaches, and therefore the best therapy for patients with MRD-positive disease after conventional therapy is enrollment in MRD-directed clinical trials. Through the incorporation of MRD as an endpoint in clinical trials and the evaluation of novel agents and combination therapies for patients with MRD-positive disease, we may get closer to achieving our goal of curing AML in the vast majority of patients.

## Data Availability

Not applicable.

## Abbreviations

Allo-HSCT: Allogeneic hematopoietic stem cell transplant
Auto-HSCT: Autologous hematopoietic stem cell transplant
AML: Acute myeloid leukemia
APL: Acute promyelocytic leukemia
BM: Bone marrow
CBF: Core binding factor
CBF AML: Core binding factor acute myeloid leukemia
CHIP: Clonal hematopoiesis of indeterminate potential
CIR: Cumulative incidence of relapse
CR: Complete remission
CRi: Complete remission without blood count recovery
CR_MRD-_: Complete remission without measurable residual disease
CRp: Complete remission with incomplete platelet recovery
ddPCR: Digital droplet polymerase chain reaction
DfN: Difference from normal
DFS: Disease-free survival
DTA: *DNMT3A, TET2*, or *ASXL1*
EFS: Event-free survival
ELN: European LeukemiaNet
FDA: Food and Drug Administration
FISH: Fluorescence in situ hybridization
HMA: Hypomethylating agent
HR: Hazard ratio
HSCT: Hematopoietic stem cell transplant
ITD: Internal tandem duplication
LAIP: Leukemia-associated immunophenotype
LFS: Leukemia-free survival
LSC: Leukemia stem cell
MFC: Multiparameter flow cytometry
MRD: Measurable residual disease
NGS: Next-generation sequencing
OS: Overall survival
PB: Peripheral blood
RFS: Relapse-free survival
RT-qPCR: Reverse transcription-quantitative polymerase chain reaction
VAF: Variant allele frequency
